# Folic acid level and preterm birth among Sudanese women

**DOI:** 10.1186/s40748-017-0065-x

**Published:** 2017-12-01

**Authors:** Manal E. Sharif, Ahmed Mohamedain, AbdelBagi A. Ahmed, Abubakr M. Nasr, Ishag Adam

**Affiliations:** 1 Faculty of Medicine, Al-Neelain University, P.O Box 12702, 11111 Khartoum, Sudan; 2 Faculty of Medicine, University of Khartoum, P.O Box 102, 11111 Khartoum, Sudan; 3 Department of Biomedical Sciences, King Faisal University, Alhasa, Kingdom of Saudi Arabia; 4 Department of Obstetrics and Gynecology, College of Medicine, King Khalid University, Abha, Kingdom of Saudi Arabia

**Keywords:** Preterm birth, Folic acid, Pregnancy, Sudan

## Abstract

**Background:**

Preterm birth (PTB) is the major health problem world-wide; there are few published studies on PTB and folic acid.

**Methods:**

The study was conducted to assess the serum level of folic acid in PTB. A case-control study was conducted at Saad Abualila maternity hospital (Khartoum, Sudan) during the period of March through December 2015. Women who delivered live singleton babies were dived in two groups; the cases were women who had PTB “delivery before completed 37weeks but after 24 weeks of pregnancy” and the controls were women who delivered at term (37–42 weeks). Medical and obstetrics history was gathered using questionnaire. Serum folic acid was measured.

**Results:**

One hundred and twelve (56 in arm of the study) women were enrolled to the study. There was no significant difference between the cases and the controls in their age, parity, hemoglobin, body mass index, education and occupation. The median (interquartile) level of folic acid was significantly lower in the cases (PTB) than the level in the controls, 4.8(2.8–8.2) vs. 9.5(8.6–12.0) ng/ml. In binary regression, folic acid level was associated with lower risk of PTB (OR=0.64; 95%=0.53–0.77, *P* < 0.001). There was a significant positive correlation between gestational age and folic acid level (*r* = 0.447, *P*<0.001).

**Conclusion:**

Thus serum folic acid level was significantly lower in women with PTB. Folic acid level was associated with lower risk of PTB.

## Background

Preterm birth (PTB) is defined by the World Health Organization as “as the delivery of the fetus before completed 37 weeks of gestation or 259 days from the first day of the last menstrual period”(‘World Health Organization. International statistical classification of diseases and related health problems. 10th ed. Geneva: [1]). PTB is a major health problem worldwide where it has been estimated that 14.9 million babies were born preterm, which estimates to 11.1% of all live births globally [2]. PTBs are the main cause of neonatal deaths and they are at a higher risk for adverse outcomes later in life e.g. cerebral palsy and learning difficulties [3]. There are two subtypes of PTB; either spontaneous (70%) “spontaneous onset of labor with intact membranes and preterm premature rupture of the membranes” or provider-indicated (iatrogenic, 30%) PTB “induction of labor or caesarean birth prior to 37 weeks of gestation” [4, 5].

Investigating the predictors for PTB is of paramount for health planners and care givers. The research could yield a helpful data to guide/implement the preventive measures for PTB. Various clinical and biological factors have been shown to be associated with PTB in different settings. [5–8]. Folate/folic acid has been studied extensively for its association with adverse pregnancy outcomes such as PTB. However, the available literature on the relationship between folate/folic acid and PTB has been inconclusive. While, some studies have found that lower levels of folate during pregnancy associated with increases the risk of PTB [9–11], other studies have failed to show association between folic acid or folate concentrations and PTB [12–14]. PTB is a major health problem in Sudan [7, 8]. The current study was conducted to assess the association between folic acid level and PTB.

## Methods

A case − control study was conducted at Saad Abualila maternity hospital (Khartoum, Sudan) during the period of March through December 2015. Saad Abualila is a tertiary hospital affiliated to the Faculty of Medicine, University of Khartoum, Sudan.

After signing an informed consent, women presented labour and had singleton babies were approached to participate in the study. The cases were women who had PTB “delivery before completed 37weeks but after 24 weeks of pregnancy, (spontaneous onset of labour or following preterm premature rupture of membranes)”. The controls were women who delivered a live born singleton baby at term (37–42 weeks). Women with intrauterine fetal death, major congenital malformation, and twins, diabetes, hypertension and antepartum haemorrhage were excluded from the cases and controls because of their known tendency to influence the risk of PTB.

From all women (cases and controls) medical and obstetrical history (age, parity, gestational age, and history of miscarriage) was gathered using a questionnaire which was filled by a trained medical officer. Gestational age was calculated from first day of the last menstrual and confirmed by ultrasound scan in the first half of pregnancy. The interpregnancy interval (IPI) was defined as the time between the woman’s previous delivery, miscarriage and the first day of the last menstrual period for the index pregnancy. Body mass index (BMI) was calculated by dividing weight in kg by height in meters squared (kg/m^2^).

Then venous blood was collected from each woman and allowed to clot in plain tubes, and the serum was stored at −20 °C until analysed for serum folate which was determined by immunofluorescent assay using IMMULITE kits according to the manufacturers’ instructions (SIEMENS Healthcare, Los Angeles, CA, USA) that we have described in our previous work [15].

A total sample of 56 participants in each arm of the study was calculated to investigate the mean difference of the folic acid level. This sample size would provide over 80% power to detect a 5% difference at α = 0.05, with an assumption that complete data might not be available for 10% of participants [16].

### Statistics

Data were entered in computer and SPSS for Windows (version 20) was used for data analyses. Continuous data were checked for normality using Shapiro-Wilk test. Data were expressed as proportions; mean (SD), median (interquartile). Mean, median (if the continuous data were not normally distributed) and proportions were compared between the cases and the controls by t-test, Mann-Whitney U and Chi-square test, respectively. Binary regression model was built where preterm delivery was the dependent variable and age, parity, education, antennal care, interpregnancy interval, hemoglobin and folic acid level were the independent variables. Odd ratios and 95% confidence interval were calculated. *P* < 0.05 was considered statistically significant. Pearson correlation was performed between gestational age and folic acid level.

## Results

One hundred and twelve (56 in arm of the study) women were enrolled to the study. The mean (SD) of their age, parity and BMI was 28.3(6.0) years, 2.4(2.3) and 26.3(4.2) kg/m^2^, respectively. Around one third (35.0, 31.3%) of these women had history of miscarriage. The majority (99.0, 88.4%) of the participants were housewives. The serum folic acid level was not normally distributed and its range from 0.9–23.0 ng/ml, the mean and median were 8.1 and 8.6 ng/ml, respectively.

There was no significant difference between the cases and the controls in their age, parity, hemoglobin, BMI, education and occupation, Table 1.

**Table 1:** comparison of the variables between the cases and controls

Variables	The cases (*n* = 56)	The controls (*n* = 56)	*P*
*The mean (SD)of*			
Age, year	28.3 (6.2)	28.3 (5.9)	0.996
Parity	2.6 (2.7)	2.1 (1.7)	0.243
Body mass index, kg/m^2^	26.2(4.3)	26.4 (4.0)	0.825
Interpregnancy interval, month	24.4(18.7)	14.9 (10.2)	0.004
Hemoglobin, g/dl	10.0 (0.9)	10.2 (1.0)	0.229
*Number (percentage)of*			
Education < secondary level	39 (69.6)	29 (51.8)	0.051
Housewives	51 (91.1)	48 (85.7)	0.278
History of miscarriage	15 (26.8)	20 (35.7)	0.208
Lack of antenatal care	10 (18.2)	6 (10.9)	0.209

**Fig. 1:**
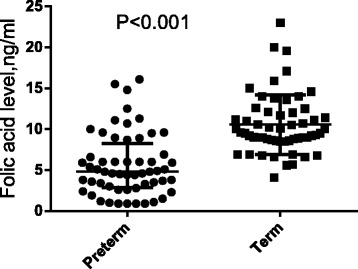
Comparing the median (interquartile) level of folic acid between the cases and the controls

The median (interquartile) level of folic acid was significantly lower in the cases (PTB) than the level in the controls, 4.8(2.8–8.2) vs. 9.5(8.6–12.0) ng/ml (Fig. 1).

In binary regression, folic acid level was associated with lower risk of PTB (OR = 0.64; 95% = 0.53–0.77, *P* < 0.001, Table 2. There was a significant positive correlation between gestational age and folic acid level (*r* = 0.447, *P* < 0.001).

**Table 2:** binary regression of the factors associated with preterm delivery

Variables	OR	95% CI	*P*
Age, years	1.03	0.94–1.13	0.485
Parity	0.93	0.72–1.20	0.589
Education < secondary level	1.26	0.68–2.31	0.454
Housewives	0.78	0.29–2.11	0.633
History of miscarriage	0.84	0.27–2.57	0.768
Lack of antennal care	1.40	0.62–3.17	0.415
Body mass index, k/m^2^	0.95	0.84–1.08	0.503
Hemoglobin level, g/dl	0.78	0.45–1.34	0.373
Interpregnancy interval, months	1.06	1.00–1.12	0.049
Folic acid level	0.64	0.53–0.77	< 0.001

## Discussion

The main finding of the current study was the significant lower level of serum folic acid in women with PTB and folic acid level was associated with lower risk of PTB. This goes with the previous studies where Chen et al., have reported that higher plasma folate concentrations were associated with a longer gestational age and associated with lower risk of PTB [9]. Likewise Furness et al., have shown that women who had a lower Red blood cells (RBCs) folate level in early (12–14 weeks) pregnancy were 5.4 times at higher risk to have PTB [11]. In a large clinical trial, it has recently been shown consumption of folic acid/ other micronutrient in the first trimester lead to a 41%–45% risk reduction for PTB [17].

In contrast to our findings, it has recently been reported that supplemental folate intake was not significantly associated with the risk of PTB [12]. Interestingly, women who received folic acid supplementation more than 8 weeks before conception were at increased risk for PTB compared with women who received no folic acid supplementation preconception [12]. In a large longitudinal study (5075 women) Yamada and colleagues failed to detect a significant association between serum folate levels during the first trimester and the risk of PTB [13]. In the later study nineteen of the 20 women with folate deficiency had no PTD. Likewise a large previous study failed to show associations of plasma folate with PTB [14]. The recent met- analysis of randomized clinical trials (included 5332 women) concluded that folic acid supplementation during pregnancy did not prevent PTB [18].

Folate has an essential role in DNA methylation and synthesis [19]. Therefore low folate concentrations can lead to unfavorable cell division and subsequently poor placentation (Scholl & Johnson, 2000). The exact mechanisms by which low folate levels are associated with PTB is not fully understood, perhaps through the homocysteine level [9, 10]. It has been shown that the women with low/normal homocysteine levels were four times at a higher risk of PTB compared with those with elevated concentrations than those with [20]. Therefore it is valuable to measure folate as well as its metabolites including homocysteine when investing folate and PTB. It is worth to be mentioned that, not only folate, or homocysteine level but also the role of genetics/mutations in pathway in the DNA repair, synthesis, methylation and its association with PTD has been observed [21, 22]. Several mutations in the methylene tetrahydrofolate reductase gene-which is responsible for the production of the active form of folate- have been shown to have an association with PTB [23, 24]. Several studies have shown that these SNPs result in an increased demand for folic acid [21, 22]. Thus these genetic factors/mutations have to be considered in the after coming research on folic acid and the associations with adverse pregnancy outcomes include PTB.

It is worth to be mentioned that in a recent Cochrane review where 17,771 women were included (in thirty-one trials) no conclusive evidence of benefit of folic acid supplementation during pregnancy on PTB was found [25]. We did not investigate cigarettes smoking in the current study. Perhaps smoking is not widely practiced among these women. Moreover, according to the Sudanese tradition, it is still difficult to investigate cigarettes smoking among females. Actually we have feared to lose the women’s co-operation or to be answered wrongly if such point was included in the questionnaire. Missing the information on smoking should be mentioned as limitation of the current study. A correlation between smoking and folate level has been reported [11, 14]. Other limitations of the study were; other confounding variables (infections) that were not addressed in the questionnaires given to the pregnant mothers, it was a small sample size (especially in the control group) single center study.

## Conclusion

The current showed that the serum folic acid level was significantly lower in women with PTB. Folic acid level was associated with lower risk of PTB.

## Abbreviations

BMI: body mass index
CI: confidence interval
OR: odd ratio
PTB: preterm birth
SD: standard devotion
